# Effect of *Gloriosa superba* and *Catharanthus roseus* Extracts on IFN-*γ*-Induced Keratin 17 Expression in HaCaT Human Keratinocytes

**DOI:** 10.1155/2014/249367

**Published:** 2014-11-10

**Authors:** Nattaporn Pattarachotanant, Varaporn Rakkhitawatthana, Tewin Tencomnao

**Affiliations:** ^1^Ph.D. Program in Clinical Biochemistry and Molecular Medicine, Department of Clinical Chemistry, Faculty of Allied Health Sciences, Chulalongkorn University, Bangkok 10330, Thailand; ^2^Center for Excellence in Omics-Nano Medical Technology Department Project, Department of Clinical Chemistry, Faculty of Allied Health Sciences, Chulalongkorn University, Bangkok 10330, Thailand

## Abstract

*Gloriosa superba* and *Catharanthus roseus* are useful in traditional medicine for treatment of various skin diseases and cancer. However, their molecular effect on psoriasis has not been investigated. In this study, the effect of ethanol extracts derived from *G. superba* leaves and *C. roseus* stems on the expression of psoriatic marker, keratin 17 (K17), was investigated in human keratinocytes using biochemical and molecular experimental approaches. Both extracts could reduce the expression of K17 in a dose-dependent manner through JAK/STAT pathway as demonstrated by an observation of reduced phosphorylation of STAT3 (p-STAT3). The inhibitory activity of *G. superba* extract was more potent than that of *C. roseus*. The Pearson's correlation between K17 and cell viability was shown positive. Taken together, the extracts of *G. superba* and *C. roseus* may be developed as alternative therapies for psoriasis.

## 1. Introduction

Psoriasis is a chronic inflammatory skin disease with hyperproliferation and abnormal differentiation of keratinocytes [1]. This disease is incurable and greatly affects patients' quality of life. The pathogenic mechanism of psoriasis suggests that keratinocytes are activated with trigger factors such as oxidative stress, drugs, smoking, and infections [2]. One important characteristic of activated keratinocytes is the alteration of keratin (K) expression from K1 and K10 to K6, K16, and K17. Especially, K17 is the only keratin induced by psoriasis-associated cytokines that has been demonstrated to play an important role in the pathogenesis of psoriasis.

K17 is classified as a type of epithelial keratins functioning in providing the epidermis integrity [3], and it is also a hyperproliferation-related keratin [4]. K17 is an interesting marker that plays a crucial role in the pathogenesis of psoriasis. In the proliferative state, K17 is highly expressed, but K17 expression decreases during the cellular differentiation process [5]. In psoriatic lesions, differentiation is impaired, thus resulting in prolonged K17 expression. The high expression of K17 is correlated with a more severe form of pathogenesis. In fact, K17 is an autoantigen with certain epitopes similar to M6 protein of streptococci. These epitopes can activate the proliferation of T cells such as T helper (Th) 1, Th 17, and IL-22-producing T cell and promote them to produce their psoriasis-associated cytokines [6–12]. Moreover, these cytokines activate keratinocytes to express and present K17, which causes a feedback loop to induce T cell proliferation and the production of psoriasis-associated cytokines. This data indicates that K17 is a link in K17/T cell/cytokine autoimmune loop [13].

*Gloriosa superba* and* Catharanthus roseus* are herbs used in traditional medicine for centuries.* G. superba* has been used to treat intestinal worms, bruises, infertility, joint pain, cancer, Kapha and Vata conditions, and skin problems [14, 15].* C. roseus* consists of many alkaloids that are useful for treatment of menorrhagia, diabetes, hypertension, and cancer [16].

In this study, the molecular effect of ethanol extracts of* G. superba *and* C. roseus* on K17 expression was investigated for use as an alternative medicine for psoriasis treatment.

## 2. Materials and Methods

### 2.1. Chemicals and Reagents

All reagents used in this study were of analytical grade. Dimethyl sulfoxide (DMSO) and ethanol were purchased from Merck (Darmstadt, Germany). 1,4-Dithiothreitol (DTT) was purchased from Bio Basic Inc. (Ontario, Canada). Phenylmethyl sulphonyl fluoride (PMSF) was purchased from United States Biochemicals (Cleveland, OH, USA). Bovine pancreatic ribonuclease A (RNAse A) was purchased from US Biological (Salem, MA, USA). Triton X-100 was purchased from SPI Supplies (West Chester, PA, USA). Propidium iodide (PI) and trypan blue solution were purchased from Invitrogen (Carlsbad, CA, USA). 4′,6-Diamidino-2-phenylindole (DAPI), piceatannol, PD98059, and Kodak processing chemicals for autoradiography films were purchased from Sigma Aldrich Co. (St. Louis, MO, USA). Dulbecco's modified Eagle medium (DMEM)/high glucose, fetal bovine serum (FBS), and penicillin-streptomycin solution (10,000 units/mL of penicillin and 10,000 *μ*g/mL of streptomycin) were purchased from HyClone (Logan, UT, USA). Recombinant human IFN-*γ* was purchased from PeproTech (Rocky Hill, NJ, USA). 3-(4,5-Dimethylthiazol-2-yl)-2,5-diphenyltetrazolium bromide (MTT) was purchased from Calbiochem (Darmstadt, Germany).

A solution of 30% acrylamide/bis-acrylamide (37.5 : 1) was purchased from Biorad (Hercules, CA, USA). Ammonium persulfate (APS) was purchased from EMD Millipore (Billerica, MA, USA). Amersham ECL Select Western blotting detection reagent and Hyperfilm ECL were purchased from GE Healthcare (Piscataway, NJ, USA). The monoclonal rabbit anti-keratin 17 (D73C7, cat#4543), polyclonal rabbit anti-phospho (p)-STAT3 (Tyr705, cat#9131), HRP-linked anti-rabbit IgG (cat#7074S), and anti-rabbit IgG conjugated Alexa Fluor 488 (cat#4412S) antibodies were purchased from Cell Signaling Technology (Beverly, MA, USA). The polyclonal rabbit anti-GAPDH (FL-335, cat#sc-25778) antibody was purchased from Santa Cruz Biotechnology (Santa Cruz, CA, USA).

### 2.2. Cell Line

HaCaT cells, an immortalized human epidermal keratinocyte cell line, were purchased from cell line service (Heidelberg, Germany). They were cultured in DMEM/high glucose containing 10% FBS and antibiotics (100 U/mL penicillin and 100 *μ*g/mL streptomycin) at 37°C in a humidified atmosphere at 5% CO_2_.

### 2.3. Plant Materials

*G. superba *and* C. roseus *were collected from the Princess Maha Chakri Sirindhorn Herbal Garden (Rayong Province, Thailand) and authenticated by Professor Dr. Thaweesakdi Boonkerd (Department of Botany, Faculty of Science, Chulalongkorn University, Thailand). The voucher specimens deposited at Prof. Dr. Kasin Suvatabhandhu Herbarium (Department of Botany, Faculty of Science, Chulalongkorn University, Thailand) were 013412 (BCU) and 013415 (BCU) for* C. roseus* and* G. suberba*, respectively. Parts used for this study were leaves and stems of* G. superba* and* C. roseus*, respectively.

### 2.4. *G. superba* and* C. roseus* Extraction

Both* G. superba *and* C. roseus *were extracted using ethanol (ratio1 : 10) at room temperature for 48 hours. The resulting products were subsequently filtered, and residues were extracted twice. Ethanol was removed by evaporation at 45°C. Final concentration of crude extracts was 100 mg/mL dissolved in DMSO as stock solutions and stored at −20°C until use. For experiments, working concentrations were prepared from the original stocks.

### 2.5. The Effect of* G. superba* and* C. roseus *Extracts on Cell Viability by MTT Assay and Trypan Blue Exclusion

Cells were seeded at 10,000 cells/well in 96-well plates for MTT and 100,000 cells/well in 12-well plates for trypan blue and incubated at 37°C for 24 hours. Having been incubated, cells were stimulated by IFN-*γ* at the final tested concentration of 1 ng/mL for 24 hours. After stimulation, media were changed. Cells were treated with* G. superba* extract (working concentrations of 0 to 100 *μ*g/mL to result in tested concentrations of 0 to 10 *μ*g/mL) or* C. roseus* extract (working concentrations of 100 to 1,000 *μ*g/mL to result in tested concentrations of 10 to 100 *μ*g/mL) or 100% DMSO as a positive control or media alone as a negative control for 48 hours. MTT stock solution (5 mg/mL) was added to each well (20 *µ*L/well) and incubated for 4 hours. In this step, formazan product was formed. Media were removed carefully and formazan product was dissolved with 150 *μ*L of 100% DMSO. Supernatant was collected by centrifugation, transferred to a new 96-well plate, and measured the absorbance of the reaction with a spectrophotometer at 550 nm. The percentage of cell viability was calculated using the formula

(1) $$\begin{array}{c} \%\;\text{cell}\;\text{viability}=\frac{\;\left({\text{Abs}}_{\text{treated}\;\text{cells}}-{\text{Abs}}_{\text{blank}}\right)}{\left({\text{Abs}}_{\text{IFN-}\gamma \text{-treated}\;\text{cells}}-{\text{Abs}}_{\text{blank}}\right)}\times 100. \end{array}$$

It should be noted that an inhibitory concentration (IC_50_) of each plant extract was calculated from the graph plotting inhibition percentage against extract concentration. For trypan blue exclusion, having been incubated with each extract for 48 hours, cells were harvested and resuspended in media. The mixture of cell suspension and 0.4% trypan blue (ratio 1 : 10) was incubated 5 min at room temperature. In the following step, cell viability was counted with a hemocytometer.

### 2.6. The Effect of* G. superba* and* C. roseus *Extracts on Cell Apoptosis and Cell Cycle

HaCaT cells were seeded at 500,000 cells/well in 6-well plates and incubated at 37°C for 24 hours. After that, cells were stimulated by IFN-*γ* at 1 ng/mL for 24 hours and treated with either* G. superba* extract (working concentrations of 0.1, 1, 10, and 100 *μ*g/mL to result in tested concentrations of 0.01, 0.1, 1, and 10 *μ*g/mL) or* C. roseus* (working concentrations of 100, 250, 500, and 1000 *μ*g/mL to result in tested concentrations of 10, 25, 50, and 100 *μ*g/mL) for 48 hours. In the following day, cells were harvested and washed by centrifugation in cold PBS at 200 g, 5 min, and 4°C. Cell suspension was added to 9 mL of cold 70% ethanol and stored at −20°C to −40°C for at least 2 hours. Cells were washed in cold PBS at 200 g, 10 min, and 4°C. Cells were resuspended in 500 *μ*L of PI/Triton X-100 staining solution: 10 mL of 0.1% (v/v) Triton X-100 in cold PBS and 2 mg of DNAse-free RNAse A in 1 mL water and 0.40 mL of 500 *μ*g/mL of PI. If RNAse was not DNAse-free, boiling of 2 mg/mL RNAse A solution for 5 min was required. Cell suspension was incubated at 37°C for 15 min, transferred tubes to ice, stored at 4°C, and protected from light. Data were acquired on flow cytometer within 48 hours.

### 2.7. Protein Expression Assays

For Western blot analysis, HaCaT cells were stimulated with IFN-*γ* at the final concentration of 1 ng/mL to induce K17 expression. After cytokine stimulation for 24 hours, they were treated with* G. superba* extract (working concentrations of 0.1, 1, 10, and 100 *μ*g/mL to result in tested concentrations of 0.01, 0.1, 1, and 10 *μ*g/mL) or* C. roseus* (working concentrations of 100, 250, 500, and 1000 *μ*g/mL to result in tested concentrations of 10, 25, 50, and 100 *μ*g/mL) or inhibitors (either piceatannol or PD98059) for 48 hours. Both inhibitors were prepared in the working concentrations of 10 and 100 *μ*M, thus resulting in the tested concentrations of 1 and 10 *μ*M. In the following day, protein extraction was carried out using 1 mM of DTT and 1 mM of PMSF in NP-40 lysis buffer. Total protein (20 *μ*g) was mixed with Laemmli buffer (ratio 1 : 1) and boiled for 5 min. Protein was separated by 10% sodium dodecyl sulfate-polyacrylamide gel electrophoresis (SDS-PAGE) and transferred onto polyvinylidene difluoride (PVDF) membranes. Membranes were blocked with 5% nonfat milk either for 1 hour at room temperature or overnight at 4°C. Membranes were incubated with K17, p-STAT3, and GAPDH primary antibodies for 1 hour at room temperature or overnight at 4°C. After incubation, membranes were washed by 1X TBS-Tween 20 (TBST) for 5 min 3 times, incubated with secondary antibodies (anti-rabbit IgG, HRP-linked antibody) for 45 min at room temperature, and washed by TBST for 5 min 3 times. Protein bands were visualized by adding the enhanced chemiluminescence detection reagent, visualized by using Amersham Hyperfilm ECL and Kodak processing chemicals for autoradiography films (Sigma Aldrich, USA). Each band was normalized against GAPDH as an internal control.

For immunofluorescence, cells were seeded in 12-well plate at 100,000 cells/well, until cell confluence was 70%, and then stimulated with IFN-*γ* for 24 hours. Media were changed, and cells were treated with either* G. superba *or* C. roseus* extract for 48 hours. Cells were washed with PBS 3 times and fixed with 4% paraformaldehyde for 20 min. Cells were permeabilized with 0.5% Triton X-100 for 30 min, and blocking with 1% FBS in PBS for 1 hour was performed. After that, cells were incubated in K17 primary antibody (1 : 200) at 4°C overnight and anti-rabbit IgG Alexa Fluor 488 (1 : 200) for 1 hour and stained with DAPI (1 : 2000 from stock solution of 5 mg/mL) for 10 min.

### 2.8. Statistical Analysis

Data were presented as the mean ± standard deviation (SD). Means were from at least three independent experiments. Data were analyzed by one-way analysis of variance (ANOVA) followed by* post hoc *Dunnett's test (*P* value < 0.05) using cytokine-treated cells as a control group.

## 3. Results 

### 3.1. Induction of K17 Expression by IFN-*γ* through JAK/STAT Pathway

**Figure 1:**
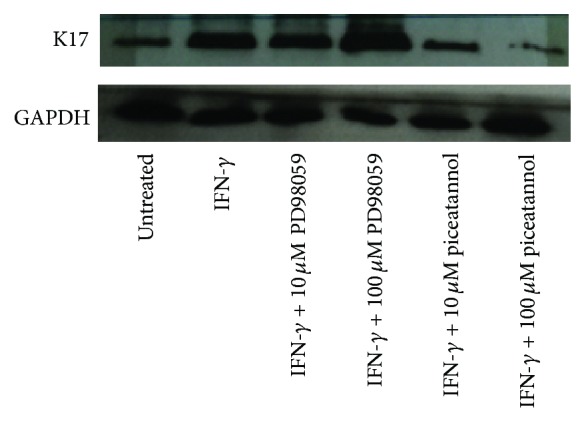
K17 expression in HaCaT when treated with IFN-*γ*, PD 98059, and piceatannol for 48 hours. In untreated HaCaT, K17 expression was found to be low, but the induced expression was detected when treated with IFN-*γ* at the final concentration of 1 ng/mL. Piceatannol could decrease K17 expression at the working concentrations of 10 and 100 *μ*M (final concentrations of 1 and 10 *μ*M), but no alteration of K17 expression was observed in PD 98059-treated HaCaT. These results implied that IFN-*γ* could induce the K17 expression through JAK/STAT pathway.

To create the psoriatic model for this investigation, we induced the expression of K17 by treating HaCaT cells with IFN-*γ*. Significantly increased K17 expression was found when HaCaT cells were treated with IFN-*γ* at the final concentration of 1 ng/mL for 24 hours. To confirm that IFN-*γ* could upregulate the K17 expression by activating the transcription factor, STAT3, we employed 2 inhibitors, piceatannol and PD98059. According to their mechanisms of action, piceatannol can inhibit STAT3 in JAK/STAT pathway, which controls the cellular response to extracellular cytokines, whereas PD98059 can inhibit MAP kinase kinase (MEK). We found that IFN-*γ* could induce the K17 expression, and the K17 protein level was significantly decreased when HaCaT cells were treated with piceatannol. Therefore, IFN-*γ* could induce the K17 expression through JAK/STAT pathway (Figure 1).

### 3.2. The Effect of* G. superba* and* C. roseus *Extracts on Cell Viability

**Figure 2:**
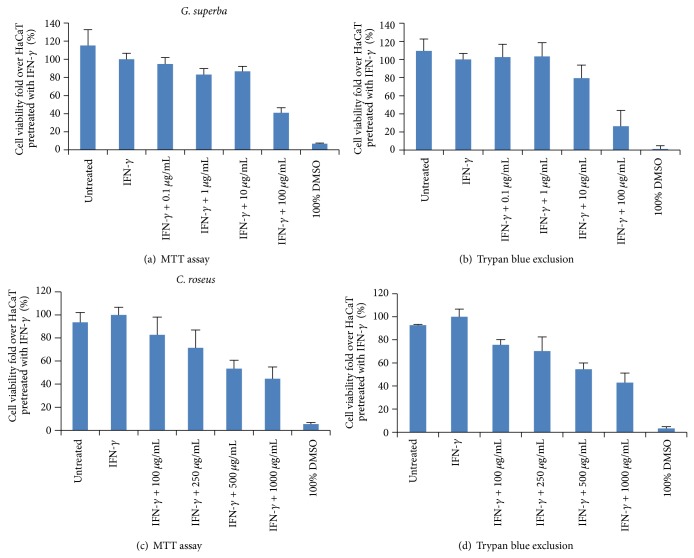
The effect of* G. superba* and* C. roseus* extracts on viability of IFN-*γ*-treated HaCaT cells. The final concentration of IFN-*γ* was applied to the cells at 1 ng/mL. Detection of cell viability was performed using MTT assay ((a) and (c)) and trypan blue exclusion ((b) and (d)). Cell viability values shown as mean ± SD were derived from 3 independent experiments. The final concentrations applied to the cells were 0.01, 0.1, 1, and 10 *μ*g/mL of* G. superba *extract and 10, 25, 50, and 100 *μ*g/mL of* C. roseus* extract.

Using the working concentrations of both plant extracts,* G. superba* extract (0.1, 1, 10 and 100 *μ*g/mL) and* C. roseus *extract (100, 250, 500, 1000 *μ*g/mL) were prepared. These concentrations tested were based on our obtained IC_50_ values, 6.09 *μ*g/mL and 74.28 *μ*g/mL for* G. superba* and* C. roseus*, respectively. Experimentally, IFN-*γ*-treated HaCaT cells were therefore treated with the final concentrations of* G. superba *extract (0.01, 0.1, 1, and 10 *μ*g/mL) and* C. roseus* extract (10, 25, 50, and 100 *μ*g/mL). We found that both plant extracts could decrease the cell viability of IFN-*γ*-treated HaCaT cells in a dose-dependent manner after treatment for 48 hours. The effect on cell viability was detected by MTT assay and trypan blue exclusion as revealed in Figures 2(a) and 2(b) for* G. superba* and Figures 2(c) and 2(d) for* C. roseus*.

### 3.3. The Effect of* G. superba* and* C. roseus *Extracts on Cell Apoptosis and Cell Cycle

**Figure 3:**
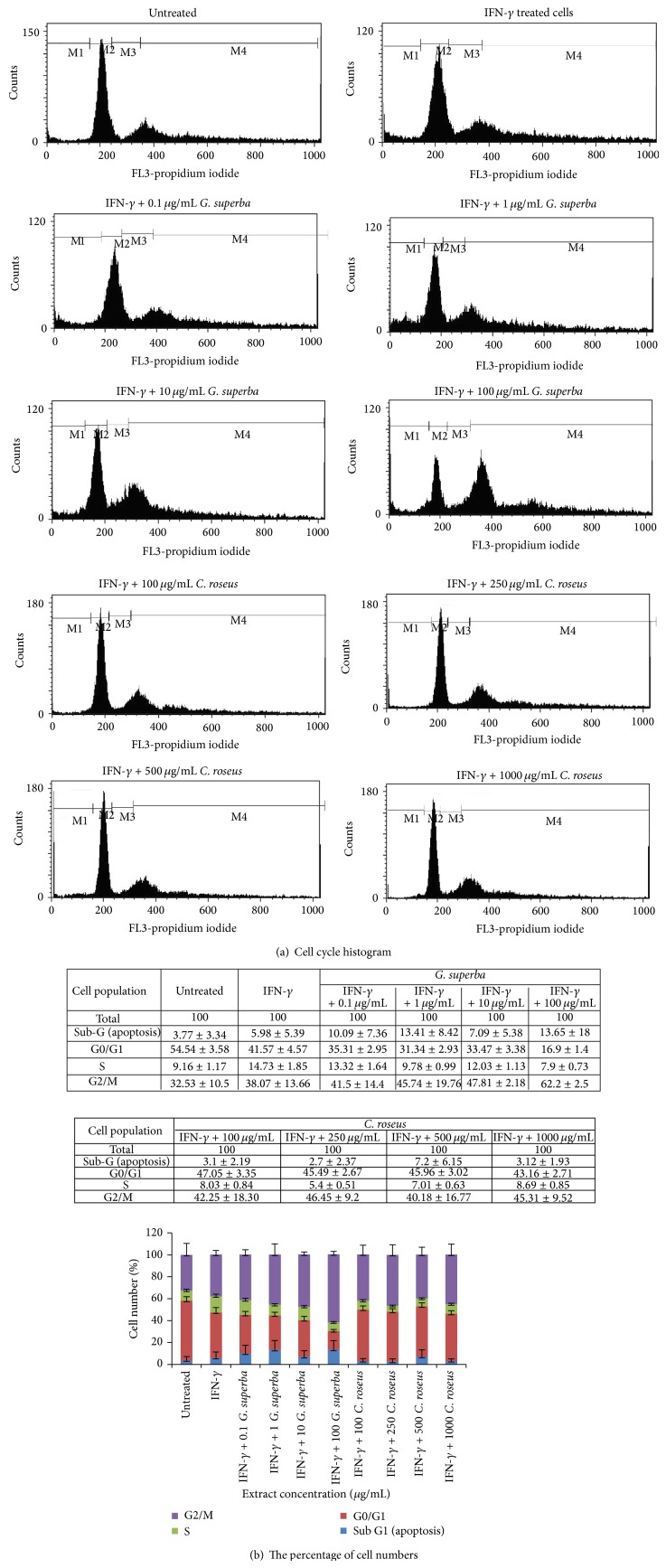
The effect of* G. superba* and* C. roseus* extracts on apoptosis and cell cycle of IFN-*γ*-treated HaCaT cells. The final concentration of IFN-*γ* was 1 ng/mL. The final concentrations of* G. superba* extract were 0.01, 0.1, 1, and 10 *μ*g/mL, and the final concentrations of* C. roseus* extract were 10, 25, 50, and 100 *μ*g/mL. Quantitative determination based on PI staining was carried out using flow cytometer. The results show cell cycle histogram (a) and the percentage of cell numbers with mean ± SD (b).

The percentage of cells in G0/G1 phase when treated with IFN-*γ* was lower than in untreated group. IFN-*γ* induced cells from resting phase (G0/G1) into cell cycle. The marked induction was observed in extract-treated groups. The percentage of cells in G0/G1 phase after treatment with such working concentrations as 0.1, 1, 10, and 100 *μ*g/mL of* G. superba* and 100, 250, 500, and 1000 *μ*g/mL of* C. roseus *extracts was found to be significantly decreased as compared with the pretreated group (*P* < 0.05) as depicted in Figures 3(a) and 3(b).* G. superba* extract at all concentrations applied in this study significantly increased the percentage of cells in G2/M phase. In particular,* G. superba* extract at the highest concentration applied to HaCaT cells in our experiment was the most potent one for inducing the cell cycle arrest at G2/M phase, while the effect of* C. roseus* extract on cell cycle with respect to all three higher concentrations was found but was not different among them. Nevertheless, the effect of* G. superba* extract on cell cycle arrest was more obvious when comparing to that of* C. roseus*.

### 3.4. The Effect of* G. superba* and* C. roseus *Extracts on K17 Expression

**Figure 4:**
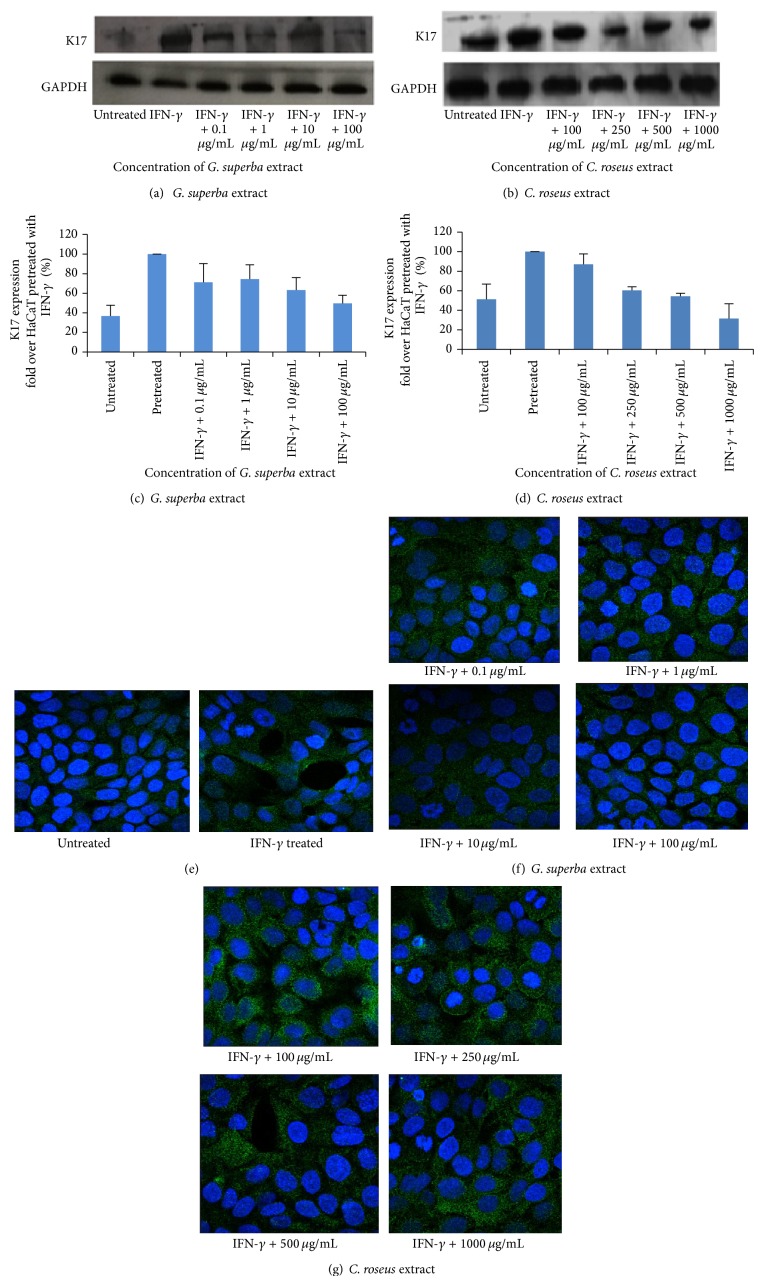
K17 expression as determined using Western blot was low in untreated group, but its overexpression was found to be induced by 1 ng/mL of IFN-*γ* (pretreated). IFN-*γ*-induced K17 expression was decreased when treated with* G. superba* extract (working concentrations of 0.1, 1, 10, and 100 *μ*g/mL) and* C. roseus* extract (working concentrations of 100, 250, 500, and 1000 *μ*g/mL) in the dose-dependent manner as shown in representative Western blot ((a) and (b)) and normalized values of K17 against GAPDH ((c) and (d)). The final concentrations of* G. superba* extract were 0.01, 0.1, 1, and 10 *μ*g/mL, while the final concentrations of* C. roseus* extract were 10, 25, 50, and 100 *μ*g/mL. The mean ± SD values of normalized K17 expression were obtained from 3 independent experiments. The effect of both extracts on K17 expression in HaCaT was also detected by immunofluorescence staining. K17 expression was in untreated and IFN-*γ*-treated groups (e). The effect of* G. superba* extract at 4 concentrations (final tested concentrations = 0.01, 0.1, 1, and 10 ug/mL, resp.) on K17 expression (f). The effect of* C. roseus* extract at 4 concentrations (final tested concentrations = 10, 25, 50, and 100 ug/mL, resp.) on K17 expression (g).

Since the K17 protein is overexpressed in psoriatic lesions and contributes to pathogenesis of psoriasis, management of psoriasis by targeting this protein may shed the light on mechanistic action of antipsoriatic drugs. In this work, we decided to investigate the effect of both extracts on the reduction of K17 expression. Using Western blot analysis, we found that* G. superba* extract (Figures 4(a) and 4(c)) and* C. roseus *extract (Figures 4(b) and 4(d)) could decrease the K17 expression in a dose-dependent manner after 48 hours of extract treatment. Furthermore, we employed an immunofluorescence confocal microscopy method as a qualitative assay to investigate the effect of both extracts on the K17 expression. Nuclei were stained with DAPI (blue) and K17 were stained with anti-K17 antibody, followed by Alexa 488 conjugated antibody (green). In untreated group, the expression of K17 was low, but the expression was elevated when HaCaT cells were stimulated with IFN-*γ* (Figure 4(e)). We found that the expression of K17 influenced by both extracts was decreased after 48 hours of the treatment (Figures 4(f) and 4(g)). These data supported our Western blot finding that both* G. superba *and* C. roseus *extracts exhibited the inhibitory effect on the K17 expression in HaCaT cells.

### 3.5. The Inhibitory Effect of* G. superba* and* C. roseus *Extracts on K17 Expression through JAK/STAT Pathway

**Figure 5:**
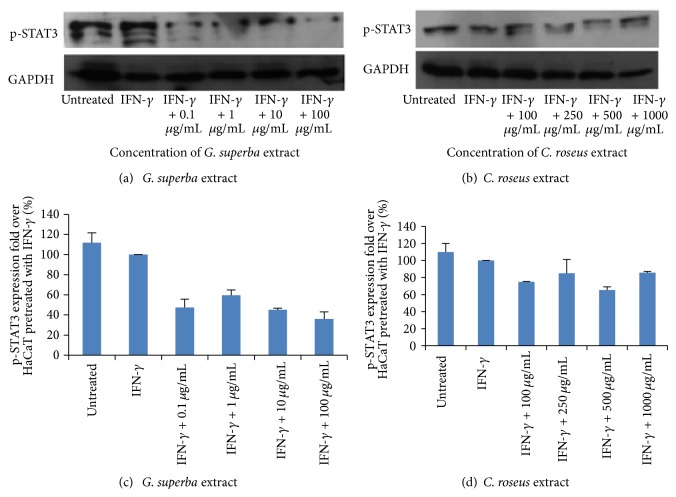
The expression of p-STAT3 was high in both untreated and IFN-*γ* groups. Its expression was significantly decreased when treated with* G. superba* extract (working concentrations of 0.1, 1, 10, and 100 *μ*g/mL) and was found to decline when treated with* C. roseus* extract (working concentrations of 100, 250, 500, and 1000 *μ*g/mL) as shown in representative Western blot ((a) and (b)) and normalized values of p-STAT3 against GAPDH ((c) and (d)). The final concentrations of* G. superba* extract were 0.01, 0.1, 1, and 10 *μ*g/mL, whereas the final concentrations of* C. roseus* extract were 10, 25, 50, and 100 *μ*g/mL. The mean ± SD values of normalized p-STAT3 expression were obtained from 3 independent experiments.

STAT3 is an important transcription factor that plays a role in K17 expression. To confirm that both plant extracts could decrease the K17 expression through JAK/STAT pathway, p-STAT3 expression was detected using Western blot. As revealed in Figure 5, the expression of p-STAT3 was significantly decreased (*P* < 0.05) when cells were treated with* G. superba* extract at the working concentrations of 0.1, 1, 10, and 100 *μ*g/mL, and a tendency of decreased p-STAT3 was observed in response to the treatment with* C. roseus* extract at the working concentrations of 100, 250, 500, and 1000 *μ*g/mL. Based on the decreased expression of p-STAT3, our results suggested that both extracts decreased the K17 expression through JAK/STAT pathway.

### 3.6. Correlation between K17 Expression and Cell Viability

K17 is known as a hyperproliferative marker, and many* in vitro* studies showed that downregulated K17 expression could inhibit cell proliferation in HaCaT. In this study, we found that* G. superba* extract (working concentrations of 0.1, 1, 10, and 100 *μ*g/mL) and* C. roseus* extract (working concentrations of 100, 250, 500, and 1000 *μ*g/mL) could lessen cell viability (Figures 2(a), 2(b) for* G. superba* and Figures 2(c), 2(d) for* C. roseus*) and could also diminish the level of K17 expression in a dose-dependent manner (Figures 4(a), 4(c) for* G. superba* and Figures 4(b), 4(d) for* C. roseus*).

**Figure 6:**
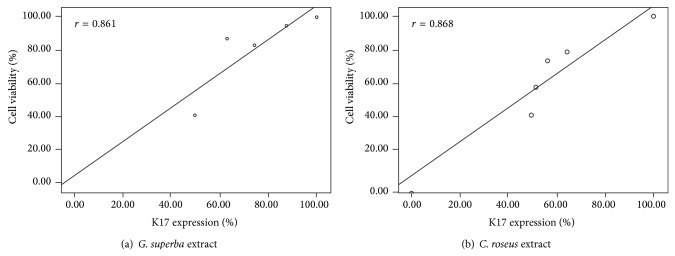
The correlation between K17 expression and cell viability showed that the level of K17 expression was positively correlated with the percentage of cell viability exerted by both extracts.

Therefore, we investigated the correlation between K17 expression and cell proliferation by Pearson's coefficient. For both* G. superba* and* C. roseus* extracts, the level of K17 expression was positively correlated with the percentage of viable cells (*r* = 0.861, 0.868, resp.) as depicted in Figures 6(a) and 6(b). This suggested that the level of K17 expression was directly proportional to the number of viable cells.

## 4. Discussion

The inhibitory effect of* G. superba* and* C. roseus* on cell division was an interesting result. Chemicals may disrupt cell division by inhibiting the polymerization of tubulin, a monomer of mitotic spindle causing cells arrest at metaphase and cell division disruption [17]. Our results indicated that both extracts may also exhibit this effect on psoriasis-associated hyperproliferation. Thus, this study investigated the role of both extracts in cell proliferation and the correlation between cell viability and the expression of K17 in IFN-*γ*-treated HaCaT cells.

IFN-*γ* is a cytokine that plays a critical role in innate and adaptive immunity for viral and intracellular bacterial infections. It is secreted by T helper 1 (Th 1), CD8+ cytotoxic lymphocytes, natural killer cells (NK), and antigen presenting cells (APCs) such as monocytes, macrophages, and dendritic cells (DCs) [18, 19]. In psoriatic lesions, the recruitment of various immune cells causes keratinocytes to be inflamed and activated by cytokines such as IFN-*γ* secretion. IFN-*γ* alters keratinization in activated keratinocytes through JAK/STAT pathway [20]. It causes high and prolonged K17 expression and K17 also plays an important role in pathogenesis of psoriasis.

Induced K17 expression in IFN-*γ*-treated HaCaT cells was alleviated by treating with piceatannol for 48 hours, indicating that IFN-*γ* mediated the K17 expression via JAK/STAT pathway. In this work, the authors explored the effect of plant extracts on K17 expression using not only Western blot technique but also immunofluorescence as a quantitative and qualitative approach, respectively. Although the modulation of K17 mRNA transcripts by both studied extracts was not assessed, we believed in our results according to the validity, accuracy, and potential of anti-K17 antibody utilized in this work. Our promising finding was based on the K17 protein level since the protein is the biomarker actually acting in the cells. Using the anti-K17 antibody for Western blot analysis, previous study conducted by Tang and collogues discovered the effect of the active fraction of* Flos Daturae *on IFN-*γ*-induced apoptosis in HaCaT cells [21].

In addition, we confirmed the molecular involvement of JAK/STAT signaling using Western blot to examine the level of p-STAT3, a critical transcription factor playing a role in K17 expression, and we found that both plant extracts could suppress the p-STAT3 expression. It should be worth addressing that STAT3 proteins activate gene in response to cytokine receptor stimulation. Upon tyrosine phosphorylation, STAT3 proteins dimerize, translocate to the nucleus, and activate certain target genes specifically such as the* cis*-element IFN-stimulated response element (ISRE), thereby originating transcription of numerous IFN-inducible genes. This transcriptional activation by STAT3 proteins requires the recruitment of coactivators such as CREB-binding protein (CBP)/p300. STAT3 proteins recognize a conserved element in the gene promoters and increase the mRNA expression of the cell cycle regulatory genes, thus modifying cell growth or transformation [22, 23]. Interestingly, there was a previous review detailing the dietary compounds as potent inhibitors of the STAT3 regulatory network [24], thus making our current finding more convincing.

In this investigation, all the concentrations of both extracts could decrease cell viability and also decrease K17 expression. Particularly* G. superba* extract exerted its effect not only in a dose-dependent fashion but also in a stronger effectiveness. The correlation between K17 and cell viability was shown positive, indicating that K17 expression was directly proportional to cell viability in IFN-*γ*-treated HaCaT cells.
